# Supplementary material for: PP2A-Mediated GSK3β Dephosphorylation Is Required for Protocadherin-7-Dependent Regulation of Small GTPase RhoA in Osteoclasts
Source: Cells. 2023 Jul 29;12(15):1967. doi: 10.3390/cells12151967 (PMC10417323; doi:10.3390/cells12151967)
Supplement: Supplementary file 1 [file cells-12-01967-s001.zip › Supplementary Legends_Revise Final.pdf]

**Supplementary Figure S1. Effect of PP2A inhibitors on osteoclast differentiation.**

$Pcdh7^{+/+}$ BMMs were treated with M-CSF (60 ng/ml) + RANKL (150 ng/ml) for three days in the presence of the indicated amount of (A) Cytostatin, (B) LB-100, and (C) Rubratoxin A. The percentage of $TRAP^{+}$ multinucleated cells (3 nuclei or more per cell) is shown (right). The scale bar represents 100 μm. Data are shown as the mean ± S.D. *** $p< 0.001$. NS: not significant.

**Supplementary Figure S2. Pcdh7 is dispensable for activation of ERK and Akt, and induction of NFATc1 in BMMs and osteoclasts.**

(A and C) BMMs derived from $Pcdh7^{+/+}$ and $Pcdh7^{-/-}$ were stimulated with M-CSF (60 ng/ml) for the indicated times, while (B and D) preosteoclasts were stimulated with RANKL (150 ng/ml) for the same duration. Whole cell lysates were then prepared and the phosphorylation status of ERK (A and B) and AKO (C and D) was analyzed by western blotting using the indicated antibodies. (E) Both $Pcdh7^{+/+}$ and $Pcdh7^{-/-}$ BMMs were cultured with M-CSF (60 ng/ml) and RANKL (150 ng/ml) for the indicated times, and whole cell lysates were prepared and subjected to western blotting using the indicated antibodies.
