# Supplementary figures and images for: PP2A-Mediated GSK3β Dephosphorylation Is Required for Protocadherin-7-Dependent Regulation of Small GTPase RhoA in Osteoclasts
Source: Cells. 2023 Jul 29;12(15):1967. doi: 10.3390/cells12151967 (PMC10417323; doi:10.3390/cells12151967)

Supplementary Figure S1

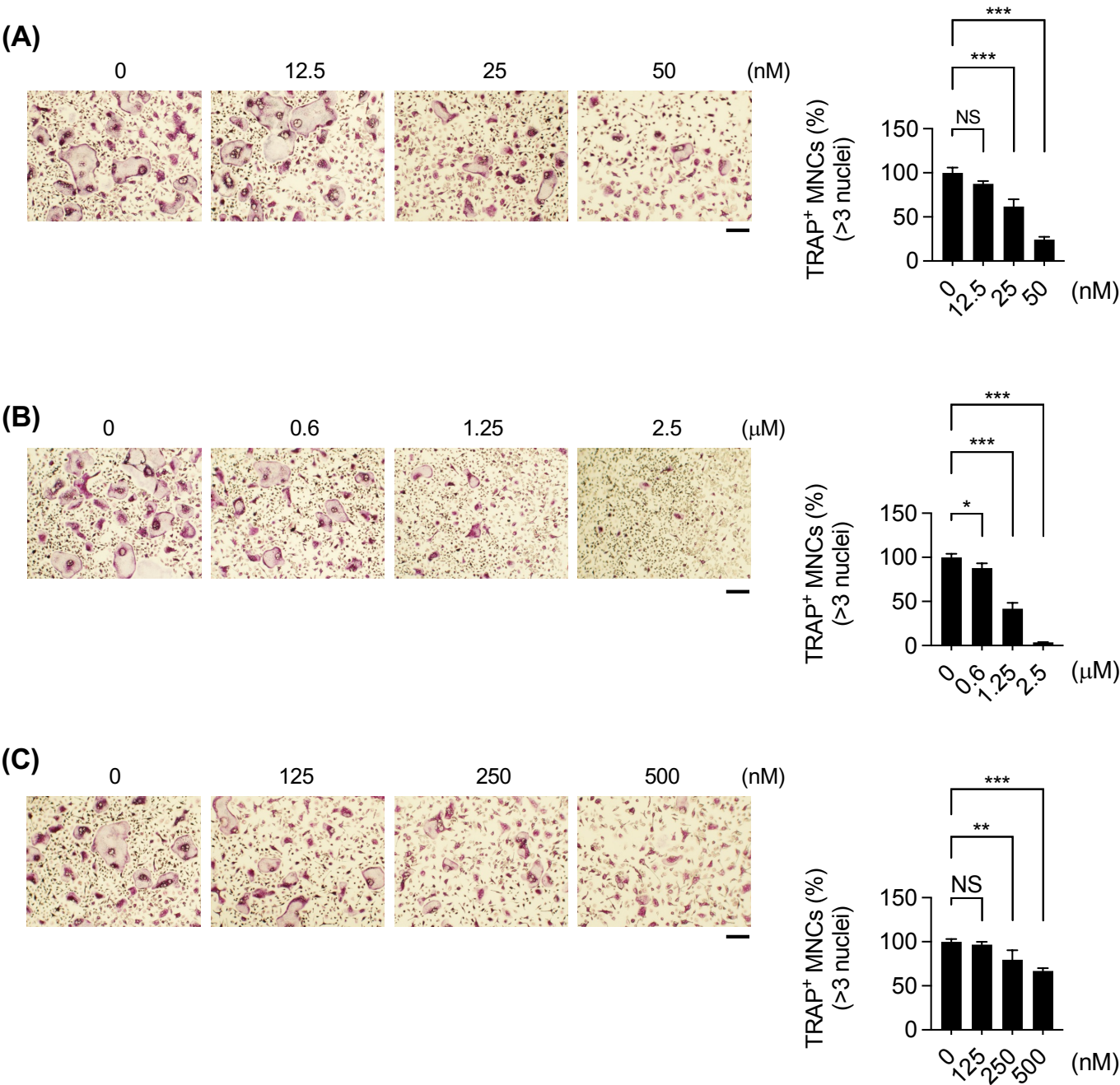

Supplementary Figure S2

(A)

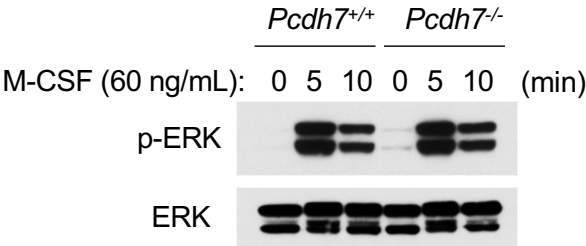

(B)

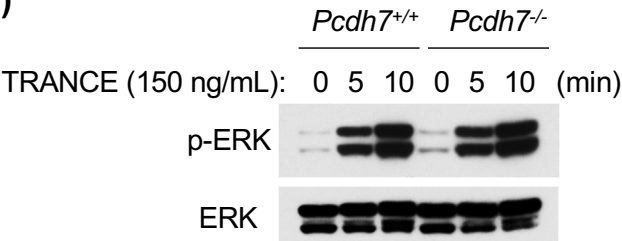

(C)

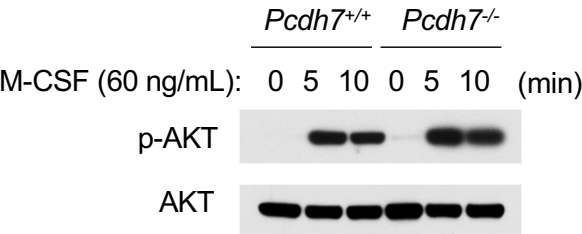

(D)

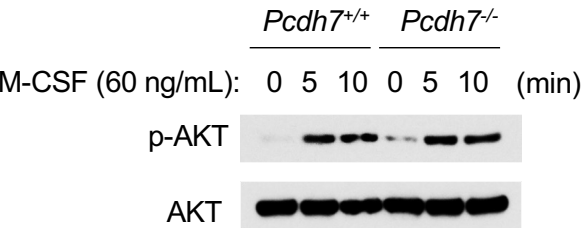

(E)

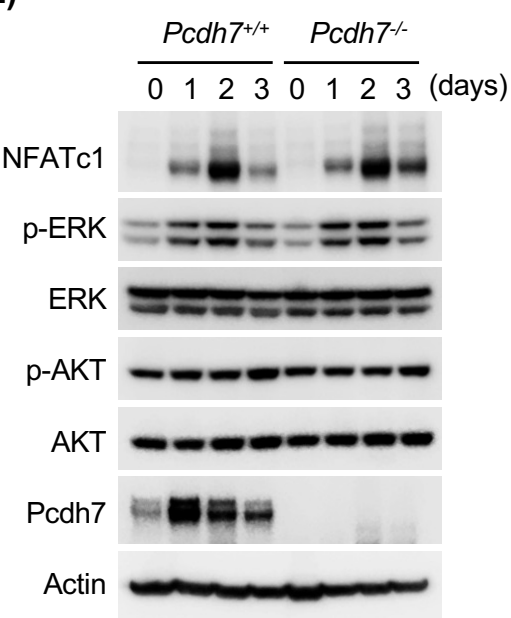

Supplement: Supplementary file 1 [file cells-12-01967-s001.zip › Supple_Revise Final.pdf]
